# Microfluidic Enrichment Barcoding (MEBarcoding): a new method for high throughput plant DNA barcoding

**DOI:** 10.1038/s41598-020-64919-z

**Published:** 2020-05-26

**Authors:** Morgan R. Gostel, Jose D. Zúñiga, W. John Kress, Vicki A. Funk, Caroline Puente-Lelievre

**Affiliations:** 1 Botanical Research Institute of Texas, Fort Worth, Texas 76107-3400 USA; 2 Laboratory of Infectious Diseases, National Institute of Allergy and Infectious Diseases (NIAID), NIH, Bethesda, MD 20892 USA; 3 Department of Botany, National Museum of Natural History, MRC 166, Smithsonian Institution, Washington, DC 20013-7012 USA; 4 Unaffiliated, Paris, France

**Keywords:** DNA sequencing, Plant sciences, Evolution

## Abstract

DNA barcoding is a valuable tool to support species identification with broad applications from traditional taxonomy, ecology, forensics, food analysis, and environmental science. We introduce Microfluidic Enrichment Barcoding (MEBarcoding) for plant DNA Barcoding, a cost-effective method for high-throughput DNA barcoding. MEBarcoding uses the Fluidigm Access Array to simultaneously amplify targeted regions for 48 DNA samples and hundreds of PCR primer pairs (producing up to 23,040 PCR products) during a single thermal cycling protocol. As a proof of concept, we developed a microfluidic PCR workflow using the Fluidigm Access Array and Illumina MiSeq. We tested 96 samples for each of the four primary DNA barcode loci in plants: *rbcL, matK, trnH-psbA*, and ITS. This workflow was used to build a reference library for 78 families and 96 genera from all major plant lineages – many currently lacking in public databases. Our results show that this technique is an efficient alternative to traditional PCR and Sanger sequencing to generate large amounts of plant DNA barcodes and build more comprehensive barcode databases.

## Introduction

*Traditional DNA barcoding and the changing landscape of molecular biology: opportunities and challenges —* The principle of DNA barcoding is rooted in the concept that a small, standardized sequence of DNA can be used to identify and discriminate among species across the tree of life^1,2^. In metazoans, the mitochondrial gene cytochrome c oxidase I (COI, cox1) has been adopted as the global standard^2^, and for fungi, the internal transcribed spacer of nuclear ribosomal DNA (nrITS) has been widely accepted as a universal barcode marker^3^.

Plant DNA barcoding has unique challenges despite the fact that it has been broadly used in traditional taxonomy, ecology, forensics, food analysis, and environmental science^4^. Since the mitochondrial genome (mtDNA) evolves slowly in plants, the levels of variation are low and insufficient to recognize species. Therefore, COI and other mtDNA markers cannot be used for plant DNA barcoding^5^. The Plant Working Group of the Consortium for the Barcode of Life (CBOL) recognizes the combination of *matK* and *rbcL* as the universal plant barcode. In large scale studies, these two loci provide a discriminatory efficiency at the species level of 72%^5^ and 49.7% respectively, and they often fail to differentiate closely related species^6^. As a result, other chloroplast regions, e.g., *trnH-psbA, trnL, trnL-F*^7–12^ and the nuclear ribosomal Internal Transcribed Spacer (ITS)^6^ are routinely used in combination with *matK* and *rbcL*^13^. Marker selection often depends on the nature of the application or research question. For instance, specimen-based studies tend to use a combination of the traditional DNA markers while metabarcoding studies aim for shorter, easy to amplify fragments (e.g., *trnL*, nuclear rDNA [ITS, 16 s], or mini-barcodes) that recover a higher number of taxa from degraded or mixed DNA samples^14–16^. Approximately twelve different primer pair combinations are necessary to amplify DNA barcode sequences for the four most widely used markers (*rbcL, matK, trnH-psbA*, and ITS) across all major lineages of vascular plants, from seedless, non-vascular plants to angiosperms (Table 1). The use of multiple loci in plant DNA barcoding increases sample handling, preparation time, and costs. However, the use of multiple markers does not always result in complete and accurate species identification^17–20^. Such limitations, along with a general lack of sampling numerous plant taxa, have contributed to an underrepresentation or absence of plant groups in most of the publicly available barcode data and genetic data repositories compared with other branches of life, thus creating a gap in the reference libraries, which are often overrepresented by flowering plants (Table 2).

**Table 1:** PCR Primer sets used in this study, with references.

Target-Specific Sequence (Forward)	Target Name	Reference
ATGTCACCACAAACAGAGACTAAAGC	rbcLa-F	Kress & Erickson, 2007
GTAAAATCAAGTCCACCRCG	rbcLa-R	Kress *et al*., 2009
TCGCATGTACCTGCAGTAGC	rbcL-724r	Cowan *et al*., 2006
ATGTCACCACAAACAGAAAC	rbcL-1f	Fay *et al*., 1997
ATGTCACCAAAAACAGAGACT	rbcL_3R-Gym	Wang *et al*., 1999
GGACATACGCAATGCTTTAG	rbcL_2F-Gym	Wang *et al*., 1999
GTTATGCATGAACGTAATGCTC	psbA3_f	Sang *et al*., 1997
CGCGCATGGTGGATTCACAATCC	trnHf_05	Tate & Simpson, 2003
TCA YCC GGA RAT TTT GGT TCG	matKGym_F1A	Li *et al*., 2011
CGTACAGTACTTTTGTGTTTACGAG	matK3F_KIM f	Ki-Joong Kim, unpubl.
ACCCAGTCCATCTGGAAATCTTGGTTC	matK1R_KIM-r	Ki-Joong Kim, unpubl.
TAATTTACGATCAATTCATTC	matK-xF	Saslis-Lagoudakis *et al*., 2008
ACAAGAAAGTCGAAGTAT	matK-MALP	Dunning & Savolainen, 2010
CGATCTATTCATTCAATATTTC	matK-390F	Cuénoud *et al*., 2002
ACCCAGTCCATCTGGAAATCTTGGTTC	matK-1RKIM-f	Ki-Joong Kim, unpubl.
TCTAGCACACGAAAGTCGAAGT	matK-1326R	Cuénoud *et al*., 2002
CCTTATCATTTAGAGGAAGGAG	ITS5a-fwd	Stanford *et al*., 2000
TCCTCCGCTTATTGATATGC	ITS4	White *et al*., 1990
GACGCTTCTCCAGACTACAAT	ITS2-S3R	Chen *et al*., 2010
ATGCGATACTTGGTGTGAAT	ITS2-S2F	Chen et al., 2010
GCAATTCACACCAAGTATCGC	ITS-C	Blattner, 1999
GGAAGGAGAAGTCGTAACAAGG	ITS-A	Blattner, 1999

**Table 2:** Comparison of the number of barcode sequences in the Barcode of Life Data System (BOLD, boldsystems.org) for major lineages of life on Earth with an estimated number of species >10,000.

Taxonomic rank	Estimated # barcode sequences^†^	Estimated # spp.	Estimated % species with barcodes
Animalia: Annelida	4,633	17,388^‡^	26.6%
Animalia: Arthropoda	228,051	1,257,040^‡^	18.14%
Animalia: Chordata	36,552	49,693^‡^	73.56%
Animalia: Cnidaria	2,674	10,203^‡^	26.21%
Animalia: Mollusca	15,557	80,000^‡^	19.45%
Animalia: Nematoda	1,493	25,033^‡^	5.96%
Animalia: Platyhelminthes	681	29,487^‡^	2.31%
Fungi	29,168	140,000^‡^	20.83%
Plantae: Magnoliophyta	65,340	352,000^§^	18.56%
Plantae: Bryophyta	1,870	20,000^§^	9.35%
Plantae: Lycopodiophyta & Pteridophyta	3,983	13,000^§^	30.64%
Plantae: Pinophyta	775	1,000^§^	77.5%

^†^Barcode of Life Data Systems, boldsystems.org, accessed 4 June 2019.

^‡^The Catalog of Life, www.catalogueoflife.org, accessed 4 June 2019.

^§^The Plant List, www.theplantlist.org, accessed 4 June 2019.

The DNA barcoding community is currently developing methods that leverage the scale of high-throughput sequencing for barcoding applications^21–26^. During a typical polymerase chain reaction (PCR), template DNA and a set of reagents are subjected to a cycle of fluctuating temperatures, producing a controlled set of enzymatic reactions^27^ that results in tens of millions of copies of a targeted DNA region. Until recently, most DNA barcoding methods follow a traditional PCR approach with a total reaction volume of 5, 10, or 15 µL followed by dideoxy chain termination (Sanger) – based sequencing. Several alternatives to traditional PCR and Sanger chemistry have been proposed over the past few years to create pooled sequencing libraries representing tens, hundreds, or thousands of samples to produce barcode sequences using 454-based^24^, Illumina^21,23^, or PACBIO^22^ high-throughput sequencing (HTS) platforms. Other approaches have turned away from the traditional DNA barcode regions and aim to sequence large portions of genomes (genome skimming) or whole genomes (organellar or otherwise^28^). Currently, some common high-throughput sequencing platforms are limited by shorter sequence read length (e.g., the longest reads from Illumina MiSeq are 600 bp) and this limitation is important for plant DNA barcoding, which leverages some loci (e.g., *matK*, approximately 1,000 bp) that exceed this read length limitation. Therefore, the barcoding community should anticipate some challenges in sequences that provide full coverage of these loci.

The advancement of high-throughput sequencing technologies has expedited the progress of plant genomics, particularly chloroplast genomics. To date, the National Center for Biotechnology Information (NCBI) organelle genome database harbors more than 1000 chloroplast genomes. These plastome data have been used mainly for studies in phylogenetics, breeding, domestication, and conservation, and have also been proposed as the plant “super-barcode”^29,30^. Complete chloroplast genomes have also shown to successfully discriminate closely related species^31–34^. Nonetheless, there are some lingering limitations to consider for the application of “super-barcodes” for broad biodiversity studies, including bioinformatic and data management challenges. Degraded samples with very low DNA concentrations in particular may pose challenges as a minimum of DNA quantity is required for this approach. McKain, *et al*.^28^ suggested that pooling more than 60 samples in a single run could exceed sequencing capacity using the greatest depth, currently-available high-throughput sequencing technology. Furthermore, whole chloroplast alignments across distantly related groups can be difficult because of the variation in gene structure, length, and organization^35^.

Coissac, *et al*.^36^ has proposed genome skimming as an expanded alternative that would recover full chloroplast genomes and rDNA, and potentially new, universal nuclear markers. These methods are still unavailable and prohibitively expensive for many research groups in regard to consumables, informatics, computational power and data storage. The key challenges to widespread adoption of genome skimming as a successor to PCR-based barcoding are summarized by the authors^36^, and include expensive and time-consuming library preparation per sample at between $25–150 (depending on library preparation kit) and up to 20 library preparations per day. On the other hand, continuing with Sanger sequencing of individual specimens, which has traditionally been the approach to generate large-scale DNA barcode libraries, could be even more expensive at large scales, in particular when multiple markers are required for a complete and reliable identification.

In this study, we introduce microfluidic enrichment (MEBarcoding) as an alternative to the methods mentioned above. MEBarcoding builds upon traditional barcoding but is more cost and time efficient, and scales easily with increasing numbers of samples and loci. Microfluidic PCR offers a highly efficient alternative to traditional PCR and Sanger sequencing. It has been used successfully in phylogenomic studies^37–39^, but its utility for plant DNA barcoding has not been assessed yet.

### Microfluidic PCR

**Figure 1:**
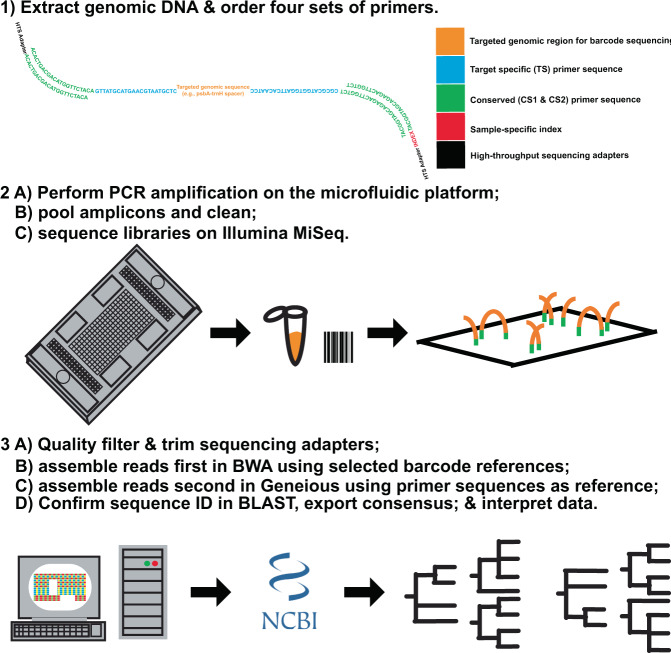
Diagram of the microfluidic PCR workflow for MEBarcoding.

The dominant commercial microfluidic instruments used for high-throughput sequencing library preparation are manufactured by Fluidigm Corporation (www.fluidigm.com, San Francisco, California, USA) and are known as the Access Array (1^st^ generation) and Juno (2^nd^ Generation). These instruments employ integrated fluidic circuits (IFC) to leverage the chemical and fluid mechanics of reagents used in the PCR at a micromolecular scale. This technology manipulates DNA samples and PCR reagents by forcing them into small volumes (0.03 µL) that interact in a modified thermal cycler. All reagents are loaded onto a single device IFC, which is approximately the same size as a 96-well PCR plate. A graphic depiction of the IFC and a simplified workflow for handling samples with this instrument is provided in Fig. 1. Actual cost and time involved with the preparation of data presented in this manuscript are provided, along with a list of reagents and product numbers for reference in Supplementary Table 1.

**Figure 2:**
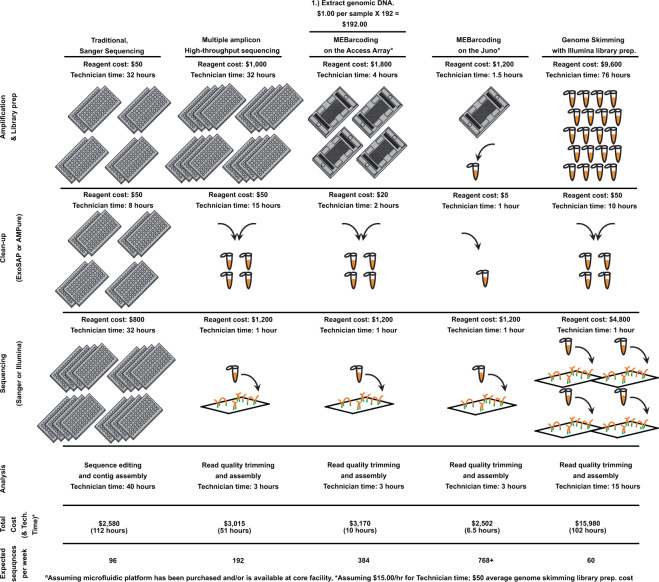
Comparison of costs from different approaches to plant barcode sequencing methods discussed in this study. Costs are estimated for a large laboratory with equipped with automated instruments for DNA extraction (Autogen) and a full time technician. For all except for Genome Skimming and the Juno, time estimates are from actual time estimates drawn from direct experience by the authors of this study and expenses reflect actual expenses from the budget used in this study, including a full time molecular technician at $15.00/hour. Multiple amplicon high-throughput sequencing (HTS) is meant to reflect methods that use a combination of traditional PCR with HTS (e.g.^21,24^). Genome skimming time and cost estimates are based upon lowest current market rates for library preparation from kits and core facilities and personal communication with genomics core facility lab technicians.

Several types of IFC are available, but the basic design features two sets of input wells that can be filled with extracted DNA samples and PCR reagents on one side and PCR primers on the other. Once loaded, the IFC is ‘primed’ by pushing the fluids through a series of microtubes that intersect, forming a central matrix of thousands of reaction chambers. The IFC used with the Access Array contains 48 DNA sample wells and 48 PCR primer wells that interact to serve as the template for 2,304 isolated combinations of sample and reagent. Using the Juno system, the scale of library preparation can be further increased with IFCs that contain 192 DNA sample wells and 24 primer wells. These instruments allow for simultaneous amplification of thousands of amplicons in miniaturized, parallel PCRs. This system is a cost-effective approach to amplify different target regions from multiple samples as it not only reduces the amount of reagent used in HTS library preparation and targeted amplification of barcode loci, but also decreases instrument and sample handling and technician time. The comparative cost, reagent use, and estimated time involved with various PCR-based methods of DNA barcoding approaches compared with those of MEBarcoding are shown in Fig. 2. The cost and time estimates provided here come from real costs involved with the data presented in this paper for both Sanger sequencing and the 48.48 Access Array microfluidic PCR approach. Other costs have been estimated based upon listed reagent costs, direct experience with these methods, and consultation with colleagues and core facilities. It is important to note that costs vary depending on equipment, location, and resources available to given laboratories – these costs are provided as estimates only, but reflect the actual cost and time associated with these methods investigated by the authors.

One technician can produce 96 barcode sequences during a 40-hour work week using traditional PCR and Sanger sequencing protocols, compared to 192 with recently published Illumina^21^ and PacBio^22^ methods. Using the MEBarcoding approach described here on the Access Array or Juno systems plant DNA barcodes could be generated for 384 or 768+ samples, respectively. An important assumption in the estimation of costs presented here is that the laboratory already has access to equipment required for MEBarcoding (approximately $40,000 for the Access Array and $100,000 for the Juno) or can utilize this instrument at one of many core facilities that make this instrument available to researchers. A laboratory working with a full-time technician, that sequences plant DNA barcodes from 96 samples per week for forty weeks would spend more than $100,000 USD using traditional Sanger sequencing protocols (see Supplementary Table 1), whereas the same number of samples could be processed in just five weeks at a cost estimated at $12,500 using MEBarcoding on the Juno system. The Juno would pay for itself in less than two years in a lab that processed a similar number of plant DNA barcodes.

### **Experimental design** limitations

Given that Illumina MiSeq v3 sequencing chemistry produces roughly 25 million reads and the broad plant DNA barcoding community has reached a reasonable consensus on four barcoding loci, we anticipate that the only limitation will be the upper threshold on the number of dual-indexed adapters available for sequencing. Assuming relatively even clustering and the ability to dual-index and pool 10,000 DNA samples onto a single MiSeq run, all four plant DNA barcodes could be sequenced with an expected 500X coverage per locus, per sample from just twenty million reads. The gain in efficiency of MEBarcoding compared with other methods ultimately is only limited by the number of samples you can pool onto a single MiSeq lane.

### **Objectives for this** method

High-throughput sequencing has radically transformed almost all facets of traditional molecular biology. At the outset of this study, we sought to determine if microfluidic PCR combined with HTS could provide a highly efficient and scalable alternative to other DNA barcoding methods. We propose MEBarcoding as a cutting-edge, high performing, and high-throughput approach to traditional DNA barcoding, based on an adapted protocol from Gostel, *et al*.^38^ using microfluidic PCR and Illumina MiSeq chemistry.

## Results

### **Microfluidic** PCR

**Figure 3:**
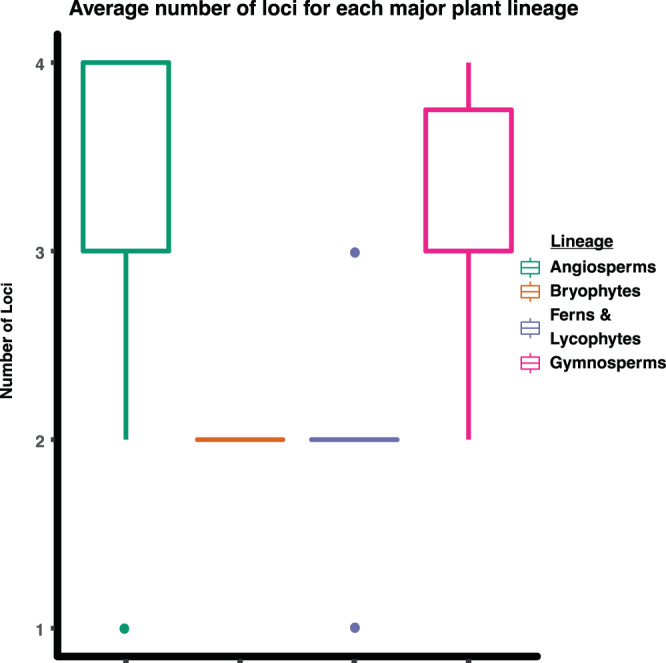
A boxplot showing the average number of plant DNA barcode loci recovered from this study, categorized by higher classification for each major lineage of land plant.

All primer pairs tested in our primer validation produced amplicons as anticipated and were used in our Access Array workflow. 96/96 samples tested for MEBarcoding produced at least one amplicon that was successfully sequenced. On average, samples in this study amplified 3.18 loci (Fig. 3). Forty-two samples amplified all four plant DNA barcoding loci, 32 amplified three loci, 19 amplified 2 loci, and 3 amplified only one locus. Sequences produced by the MEBarcoding approach were compared to sequences produced by Sanger sequencing and are summarized in Table 3.

**Table 3:** Comparison of PCR amplification and sequencing success rate from the traditional PCR and Sanger Sequencing approach and the MEBarcoding results from this study.

Marker	# successful sequences from MEBarcoding	# successful sequences from Sanger sequencing	Average pairwise distance between MEBarcoding & Sanger
*rbcL*	91/96	94	99.59%
*matK*	58	71	99.3%
*trnH-psbA*	83	88	97.56%
ITS	64	67	95.57%

When available, sequences from both approaches were compared and a pairwise identity score is provided.

### **Sequence read** characteristics

**Figure 4:**
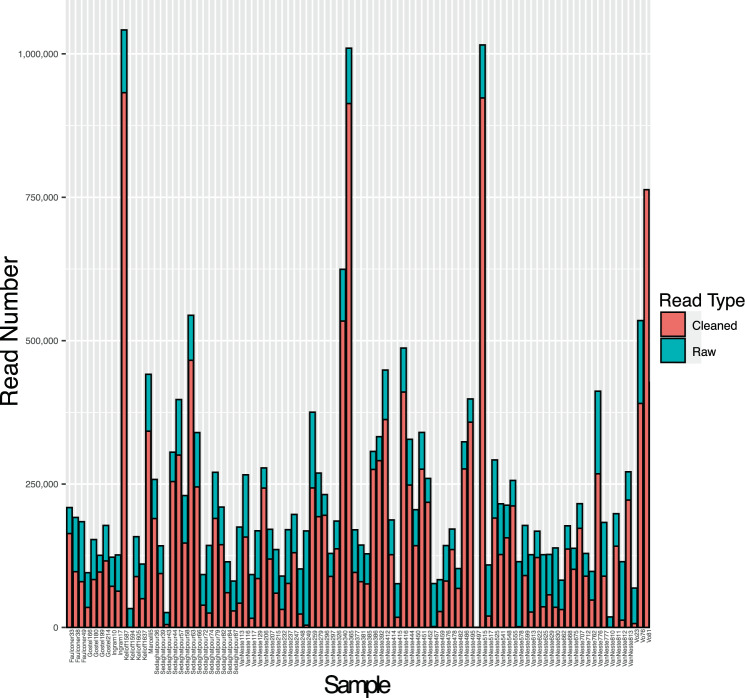
A barplot showing the relative number of sequencing reads generated for each of the 96 plant species sampled in this study. Blue and Red bars correspond to the number of reads from raw and cleaned sequence data, respectively.

**Figure 5:**
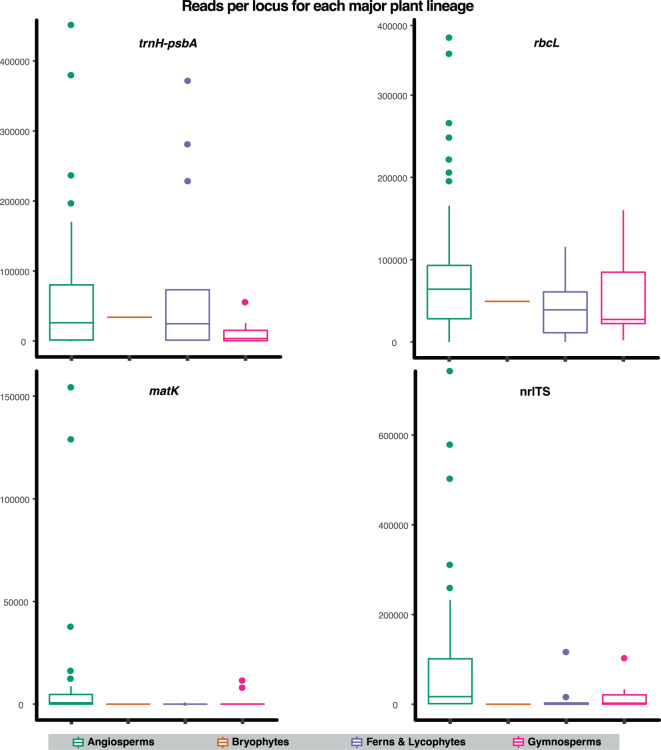
Boxplots showing the average number of sequence reads per sample from MEBarcoding (microfluidic PCR) for each marker employed in this study, organized by locus.

Our dataset yielded 15,737,842 sequence reads following sequence quality filtering and adapter trimming. Sequencing produced an average of 163,936 reads per sample, with a minimum of 119 and a maximum of 932,436 reads (Fig. 4). All raw sequence reads are deposited in the National Center for Biotechnology Information Sequence Read Archive (NCBI SRA:  PRJNA389125). Statistics for each marker are summarized in Fig. 5 and Table 3. After clean up, only three samples failed to produce more than 1,000 reads (Table S1). The number of sequence reads was uneven across loci. Our results reveal amplification bias toward certain loci (*rbcL* and *trnH–psbA*) and samples using this method. On average, we recovered close to 229,000 reads (163,935 after adapter and quality trimming) and 3.1 loci from each sample. ITS and *matK* were problematic in bryophytes, ferns, and lycophytes; and *trnH-psbA* in Gymnosperms (Fig. 5). The underrepresentation of these loci in such groups is not particularly surprising as a growing body of literature has reported similar results for these three markers^40–44^. The maximum sequencing depth was from nrITS, with an average of 78,518 reads per sample, followed by *trnH-psbA* and *rbcL* close behind with 62,158 and 72,131, respectively (Fig. 5). The lowest sequencing depth came from *matK*, which averaged 8,489 reads per sample (among samples for which this marker was successfully assembled).

### **Barcode sequence comparison and** validation

On average, sequences produced by MEBarcoding were 98% similar to their Sanger sequence counterpart. *rbcL*, *matK*, *trnH-psbA*, and ITS each shared 99.59%, 99.3%, 97.56, and 95.57% average pairwise identity, respectively. Twenty-two sequences produced with MEBarcoding were removed from the analyses after comparison to BLAST results produced dubious results and are not included in the results we report here. Of the sequences that were excluded, ten did not produce data with Sanger sequencing either, nine produced very low number of reads (<100; <50X coverage), and three were of poor quality (high proportion of ambiguities) (Table S1). Two hundred and ninety-six sequences were retained with MEBarcoding: 91 *rbcL*, 58 *matK*, 83 *trnH-psbA*, and 64 nrITS, whereas the Sanger-sequenced counterparts for the same DNA samples resulted in 320 amplicons: 94 *rbcL*, 70 *matK*, 88 *trnH-psbA*, and 67 nrITS (Table 3). The number of loci produced for each sample from MEBarcoding and Sanger barcoding methods is provided in Table 3 and a summary of statistics (number of cleaned sequence reads per sample, per locus and pairwise identity between MEBarcoding and Sanger produced sequences) describing differences between sequences for all samples produced by each method is provided in Table S1.

## Discussion

Like most methods at the cutting edge of molecular biological technology, MEBarcoding has enormous and rapidly evolving potential. Results in this study show the capacity of a first-generation microfluidic instrument, the Fluidigm Access Array. With the ability to prepare 192 barcode libraries in approximately 5 hours on the Juno instrument, output can be increased fourfold. We estimate that a single lane of Illumina MiSeq using v3 chemistry could produce sufficient barcode sequence data for approximately 500 pooled samples (10 × 48.48 IFCs or 2 × 192.24 IFCs). Furthermore, the proof of concept and workflow provided here demonstrates that MEBarcoding is not only practical for plant DNA barcoding studies, but potentially any DNA barcoding study that targets multiple regions, particularly the rapidly growing field of metabarcoding^45^.

One of the biggest challenges to any DNA barcoding study is the continued lack of barcode reference sequences for all taxa of interest in existing databases^4^ (Table 2). However, as barcode databases expand, the capacity of DNA barcoding to accurately identify an unknown sample increases. MEBarcoding would facilitate and accelerate the expansion of such databases by generating sequence data for multiple loci in a single reaction. If sampling depth was increased by 100-fold – to 960 samples – on this platform (and if sequence read efficiency scaled linearly), the expected average coverage would be 84X from even the lowest performing plant DNA barcode locus (*matK*). Therefore, we expect this method would scale remarkably well with increased sampling despite the difference in relative sequencing success between some markers.

### **Challenges** and **limitations**

In many ways, the hindrances to MEBarcoding are similar to those for traditional PCR-based plant DNA barcoding; but the cost and time necessary to carry out research projects with high sample volumes is lower using the MEBarcoding approach. This technique does, however, involve two limitations that are unique and involve (1) a high initial equipment cost, (2) comparatively lower sequencing success to Sanger methods. First and perhaps the most immediate limitation is the high initial cost if a researcher does not have access to an instrument capable of performing microfluidic PCR. The instruments discussed in this manuscript (Access Array and Juno) come with a price tag between $40,000 and $100,000, respectively, which may be a prohibitively expensive upfront cost; however, if this is cost-prohibitive there are several core facilities in the United States that offer library preparation services with one of these instruments (e.g., ibest, https://www.ibest.uidaho.edu/). Second, overall sequencing success is marginally lower than that of Sanger sequencing according to results in Table 3 (95% vs. 97% for *rbcL*, 60% vs. 74% for *matK*, 87% vs. 92% for *trnH-psbA*, and 67% vs. 70% for ITS). It should be noted that all Sanger sequences were run twice, in the event that a first sequence attempt failed (~10%, Zúñiga, pers. comm.); however, MEBarcoding sequences were not rerun. The benefits of this system should be balanced with the costs for researchers to ensure their program is leveraging the cost-effectiveness of MEBarcoding with high-volume sample processing.

As with traditional plant DNA barcoding, longer loci, such as *matK*, present several limitations, which we address in three ways. First, longer PCR amplicon targets (i.e., *matK*, >1,000 bp) are well-known to amplify at a lower efficiency than shorter amplicons^46^. Our results suggest that, on average, *matK* produced fewer sequence reads (86%, 89%, and 88%) than the comparatively shorter *rbcL*, *trnH-psbA*, and nrITS, respectively. Second, the Illumina v3 MiSeq kit produces sequence reads of up to 300 bp, which is insufficient to cover the full length of the *matK* region (>1,000 bp). Therefore our consensus sequence assemblies for this marker contain a gap of approximately 200–300 bp. Third, recovering *matK* sequences has been regarded as a challenge in certain plant lineages for various reasons, particularly among ferns^43^. Alternative HTS platforms (e.g. Pacbio SMRT) could be better suited to build plant DNA barcode libraries due to the length of the *matK* region. Pacbio recently was used to sequence barcode sequence libraries for COI^22^. Combining Pacbio with MEBarcoding could help determine whether the longer sequence read length provided by this single molecule, real time (SMRT) sequencing approach^47^ (up to 60 kb) can improve the recovery success of all four plant DNA barcode loci (especially *matK*) examined in this study. Bias toward short amplicon fragments may continue to be a hindrance to high-throughput plant DNA barcoding because sequencing libraries generated during amplification are inherently skewed toward a higher density of shorter amplicon targets. If *matK* remains recalcitrant in high-throughput approaches, we suggest removing this locus from the core list of plant DNA barcode loci.

### Future directions and scaling the experimental design

The continuous growth and implementation of DNA barcoding is dependent upon the progressive contribution of sequence data for diverse taxa across all branches of the tree of life. As reference libraries expand, our ability to rapidly identify unknown samples will continue to provide an unparalleled tool for biodiversity research. MEBarcoding offers a cost and time efficient alternative for large-scale, multi-loci DNA barcoding, and allows for the simplification and acceleration of other large scale diversity studies that were previously beyond the capacity for traditional PCR and first generation sequencing methods. The potential for MEBarcoding to help grow and enhance the quality of plant DNA barcode reference libraries cannot be understated. This technique provides a template for massive, worldwide plant DNA barcoding that can help advance the priorities of the global barcoding community rapidly and therefore facilitate downstream applications that depend upon diverse and high-quality barcode reference sequence databases.

Besides expediting the construction of plant DNA barcode libraries, we envision MEBarcoding as particularly valuable for DNA metabarcoding and its application to biodiversity research^48^; wildlife forensic identification^49^, and food and natural products detection and authentication^4,16,50,51^, see Kress, 2017 for review of plant DNA barcoding applications. It would also be an effective method for evaluating the utility of different DNA barcodes for undersampled taxa (e.g. algae and other microbial eukaryotes^52^ and optimizing DNA barcoding primers for a set of taxa^53^). We anticipate continuous refinement of the MEBarcoding approach, which in addition to high efficiency, is also flexible and can accommodate new and/or custom primer sets as new loci are developed. This could provide a means to sequence entire plastomes, as has been suggested for “super barcodes” (Kane & Cronk, 2008), combining an array of PCR primer combinations to target the entire chloroplast genome, similar to the “long-PCR” approach presented by Uribe-Convers *et al*. (2014).

One of the most exciting uses of high-throughput sequencing in DNA barcoding is the possibility of sequencing a community of organisms from a single sample. This technology has offered a mechanism to scale up the size of DNA barcoding studies from single organisms, to a collective, community sample. DNA metabarcoding emerged as a method describing “high-throughput, multispecies identification” from environmental samples^45^. It has been shown as an effective tool to accurately recover multiple levels of diversity from either known, long-term study sites^54^ or community samples also characterized using visual identification^55^. Moreover, metabarcoding has been applied to address research questions that were previously intractable, such as niche partitioning in the diets of large mammalian herbivores through fecal samples^56^; identification of plant visitation networks through pollen diversity carried by bee pollinators^57,58^; marine benthic community diversity revealed by artificial reef structures^59^; similar studies on terrestrial soil fungal communities^60^; and invertebrate seagrass communities^55^, among others.

Metabarcoding, like its counterpart in community ecology, can be complex and costly in implementation, but these issues have become increasingly surmountable. We identify three principle challenges to metabarcoding: first, insufficient global databases for barcode sequence-based identification; second, repeatability of community studies; and third, sufficient affinity of primers for diverse community samples at varying scales of taxonomic diversity. We anticipate the first challenge, regarding sequence databases to be resolved over time as barcoding databases are continuously updated. The second challenge, posed by repeatability in samples is unique and will not necessarily be ameliorated by growth in scientific knowledge; several studies have attempted to overcome this by advocating for multiple replicates per sample in their metabarcoding studies. Multiple replicates have been used to demonstrate robustness in several recent studies^59,61,62^, however best practices should be a priority for the metabarcoding research community. For each replicate, reagent and handling costs increase, respectively and as the number of samples increases, these expenses can quickly render large scale studies cost-prohibitive. The third challenge, posed by primer specificity, depends upon the scale of the research project being undertaken. A recent attempt by Brown *et al*. (2016) to improve fungal metabarcoding has optimized primer combinations using microfluidics on the Fluidigm Access Array platform. A strategy employing a set of optimized primer sets that target a broad range of eukaryotic and prokaryotic lineages on microfluidic PCR platforms will allow for improved scaling of metabarcoding approaches that reveal community diversity across the tree of life. Such an approach follows a global need for improved standards^63,64^ that seek to unify best practices by implementing proven, standardized barcode markers that are being utilized in molecular biodiversity reference databases^5^. Each of these challenges can be addressed by MEBarcoding, reducing reagent use and handling time by an order of magnitude.

The challenges posed to global biodiversity research are multifaceted and require a coordinated research effort that not only maintains best practices, but also continually revisits them to incorporate continuously novel discoveries and technologies. MEBarcoding is one such technology that has the capacity to reduce costs and sample processing time and therefore transform the way DNA barcodes are sequenced for comparative studies of diversity at both the species and community scale.

## Materials and methods

### Taxonomic sampling

96 samples were selected from across the vascular plant tree of life (Supplementary Table 2) representing 78 families and including all major lineages of land plants (e.g., Bryophytes, Ferns and Lycophytes, Gymnosperms, and Angiosperms). All samples were collected as per Funk *et al*. (2017) as part of the Global Genome Initiative for Gardens program^65^, and are publicly searchable through the Global Genome Biodiversity Network^66^ (GGBN) web portal (http://www.ggbn.org/ggbn_portal/). These have been sequenced using traditional PCR and Sanger chemistry for plant DNA barcode loci in another publication^67^. This set of samples was selected specifically so that a direct comparison could be made between traditional barcode sequencing methodology and the MEBarcoding approach presented here and for representation across plant groups.

### DNA Extraction

All tissue sampling and DNA extractions were done at the Laboratories of Analytical Biology (LAB) facilities at the National Museum of Natural History in Washington, DC and at the Museum Support Center in Suitland, MD. Silica-preserved leaf tissues were placed in a 96-well plate preloaded with glass and ceramic beads, which was then placed in a FastPrep 96 instrument (MP Biomedicals, Santa Ana, CA, USA) for tissue disruption. Whole genomic DNA was isolated using an AutoGenprep 965 (Autogen, Holliston, MA, USA) automated extractor following the manufacturer’s protocol for plant tissue.

### PCR primer design and validation

The twelve different PCR primer pairs employed in this study were selected from the literature and from the BOLD primer dataset platform (http://www.boldsystems.org/index.php/Public_Primer_PrimerSearch) to target the four most commonly cited plant DNA barcoding loci (*rbcL*, *matK*, *trnH*-*psbA*, and ITS) (Table 1). Primers were validated according to the primer validation protocol described in the Fluidigm User Guide (Fluidigm PN 100–3770, San Francisco, California, USA), using the FastStart High Fidelity PCR System (Sigma-Aldrich). Amplicons were visualized using gel electrophoresis on an agarose gel (1.5% agarose, 90 V for 45 minutes). All primer pairs used in the validation protocol produced amplicons as anticipated and were retained for use on the Access Array.

### Microfluidic PCR amplification for library preparation and clean-up

Microfluidic PCR amplification was carried out on a Fluidigm Access Array at the Center for Conservation Genomics at the Smithsonian Institution’s Conservation Biology Institute (Washington, District of Colombia, USA) and followed the protocol for “4-Primer Amplicon Tagging 48.48 Access Array IFC” outlined in the Fluidigm Access Array User Guide (Fluidigm PN 100–3770, San Francisco, California, USA). Amplicon libraries from all 96 samples were pooled into two tubes (48 samples each) and cleaned using the Agencourt AMPure XP kit (Beckman Coulter, Inc., Brea, California, USA). Prior to sequencing, the library was quantitated using a Qubit fluorometer and diluted to 2 µmol/µL with DNA Suspension Buffer (TekNova T0221, Hollister, California, USA).

### High-throughput sequencing

The cleaned, pooled library was sequenced on an Illumina MiSeq (Illumina, Inc. San Diego, California, USA) instrument using v3 chemistry (2 × 300, 600-cycle) sequencing kit at LAB. Custom sequencing primers, from Exiqon, Inc. (Woburn, Massachusetts, USA), were used in the sequencing reactions according to the Fluidigm Access Array User Guide (Fluidigm PN 100–3770).

### Sanger sequencing

Sanger sequence data were generated for each of the 96 samples utilized by Zúñiga *et al*. (2017). This study produced 62, 67, 79, and 83 barcode sequences for ITS, *matK*, *trnH-psbA*, and *rbcL*, respectively. Prior to this study, plant DNA barcode sequences had never been produced for these loci in these taxa. When a sequence was generated by both methods, we compared sequences produced by the MEBarcoding approach to the previously generated sequences using pairwise distances. When novel barcode sequences were produced by the MEBarcoding approach, but not the traditional approach, we confirmed the sequence identity based on comparative metrics from a nucleotide BLAST search (when available).

### Sequence read processing

Illumina MiSeq reads were quality trimmed and filtered using a custom script in CutAdapt 1.8.1^68^. This script is available in Dryad (10.5061/dryad.ps8ng8g). Reads were filtered according to the method outlined by Gostel, *et al*.^38^, which includes removal of ends with quality scores <Q20, removal of reads shorter than 60 bp, trimming poly-N tails ≥ 6 bp, and trimming all Illumina adapter sequences from the ends of reads. Mapping of sequence reads followed two approaches; first using a combined approach with the software package BWA v 0.7-17^69^ and – for read files that did not successfully map to a reference using the BWA approach – using the “Map to Reference” feature in the software package Geneious v 11.1.2^70^.

Read mapping in BWA was performed using the BWA-MEM setting^71^. We defined a reference index (using the “index” command) from a set of barcode reference sequences representing phylogenetically similar taxa, including 20 taxa from across the plant tree of life (Reference sequence dataset in Dryad, 10.5061/dryad.ps8ng8g). All SAM files generated by BWA were converted to BAM files, using a script in samtools (Dryad, 10.5061/dryad.ps8ng8g) and the resulting BAM files were imported into Geneious to visualize and review each locus assembly. Not surprisingly, the BWA approach was not able to reliably assemble 100% of our sequenced barcode data. In some cases, it is likely that a barcode sequence was not able to be amplified and sequenced for one or more markers in some samples (see *Discussion*); however, in other cases it may be that read mapping was not possible because the BWA algorithm is not optimized for short reference sequences (i.e. 500–600 bp barcode sequences), but rather complete genome reference sequences. To rescue barcode sequence data from samples that were not successfully assembled with BWA, we used a second approach, described below. All BAM files generated by the BWA assembly were imported into Geneious v 11.1.2 (henceforth, “Geneious”) and a 50% (strict) majority consensus file was exported as a .fasta file for each locus. Before computing the consensus sequence, we deleted all annotations that corresponded to soft-clipped sequences.

Any sample (and corresponding barcode locus) that did not produce assemblies in BWA was analyzed separately in Geneious using the “Map to Reference” feature. Cleaned.fastq read files were loaded into Geneious and grouped into a folder for each sample. In each folder, we also imported a.fasta reference sequence file that contained nucleotide sequences for each of the four barcode loci (or their primer sequences) included in this study. Assembly was carried out using the “Map to Reference” option in Geneious using default settings under the “highest sensitivity” option. 50% (strict) majority consensus sequences resulting from reads that mapped to reference sequences using this approach (but were not successful using the BWA approach) were then exported as a.fasta file and saved for subsequent analysis with the corresponding BWA-assembled consensus sequences for each locus.

### Comparison of Sanger sequencing and the MEBarcoding approach

The consensus reads obtained through MEBarcoding were loaded into Geneious, and BLAST searches were conducted using the blastn algorithm and limiting search results to 100 hits. All barcode sequences previously generated for these samples through Sanger sequencing were already discoverable on GenBank at the time the BLAST searches were carried out. Pairwise distances between sequences generated through the Sanger and MEBarcoding approaches were recorded if both were available; as well as the taxonomic level of the BLAST match; and whether the corresponding Sanger sequence was among the top ten BLAST results or not among the results altogether (Supplementary Table 2).

## Supplementary information

Supplementary information.

Supplementary Table 1

Supplementary Table 2

## Data Availability

All read data has been stored in the NCBI Sequence Read Archive PRJNA389125. All scripts used to analyze and assemble sequence data as well as a.fasta file containing the consensus sequence for each sample – organized by locus - generated in this study have been submitted to Dryad (10.5061/dryad.ps8ng8g). Complete voucher information (including locality, genbank accession numbers, and biorepository ID) for each sample accession used in this study is provided in Supplementary Table 2.
