# Author Correction: Microfluidic Enrichment Barcoding (MEBarcoding): a new method for high throughput plant DNA barcoding

**DOI:** 10.1038/s41598-020-69598-4

**Published:** 2020-07-22

**Authors:** Morgan R. Gostel, Jose D. Zúñiga, W. John Kress, Vicki A. Funk, Caroline Puente-Lelievre

**Affiliations:** 1 Botanical Research Institute of Texas, Fort Worth, Texas 76107-3400 USA; 2 Laboratory of Infectious Diseases, National Institute of Allergy and Infectious Diseases (NIAID), NIH, Bethesda, MD 20892 USA; 3 Department of Botany, National Museum of Natural History, Smithsonian Institution, MRC 166, Washington, DC 20013-7012 USA; 4 Unaffiliated, Paris, France

Correction to: *Scientific Reports* https://doi.org/10.1038/s41598-020-64919-z, published online 26 May 2020

This Article contains a typographical error in the Materials and Methods section under the subheading ‘Microfluidic PCR amplification for library preparation and clean-up’ where,

“Microfluidic PCR amplification was carried out on a Fluidigm Access Array at the Center for Conservation Genomics at the Smithsonian Institution’s Conservation Biology Institute (Washington, District of Colombia, USA) and followed the protocol for “4-Primer Amplicon Tagging 48.48 Access Array IFC” outlined in the Fluidigm Access Array User Guide (Fluidigm PN 100–3770, San Francisco, California, USA).”

should read:

“Microfluidic PCR amplification was carried out on a Fluidigm Access Array at the Center for Conservation Genomics at the Smithsonian Institution’s Conservation Biology Institute (Washington, District of Columbia, USA) and followed the protocol for “4-Primer Amplicon Tagging 48.48 Access Array IFC” outlined in the Fluidigm Access Array User Guide (Fluidigm PN 100–3770, San Francisco, California, USA).”

