# Supplementary material for: Microfluidic Enrichment Barcoding (MEBarcoding): a new method for high throughput plant DNA barcoding
Source: Sci Rep. 2020 May 26;10:8701. doi: 10.1038/s41598-020-64919-z (PMC7250904; doi:10.1038/s41598-020-64919-z)
Supplement: Supplementary file 1 — Supplementary information. [file 41598_2020_64919_MOESM1_ESM.docx]

**Supplementary information**

**Microfluidic Enrichment Barcoding (MEBarcoding): a new method for high throughput plant DNA barcoding**

*Running title: High throughput plant DNA barcoding*

Gostel, Morgan R.^1^

Zúñiga, Jose D. ^2^

Kress, W. John ^3^

Funk, Vicki A. ^3^

Puente-Lelievre, Caroline

^1^Botanical Research Institute of Texas, Fort Worth, Texas 76107-3400, USA

^2^Laboratory of Infectious Diseases, National Institute of Allergy and Infectious Diseases (NIAID), NIH, Bethesda, MD, 20892 USA

^3^Department of Botany, National Museum of Natural History, MRC 166, Smithsonian Institution, Washington, DC 20013-7012, USA
